# Association of *KRAS* Variant Subtypes With Survival and Recurrence in Patients With Surgically Treated Intrahepatic Cholangiocarcinoma

**DOI:** 10.1001/jamasurg.2021.5679

**Published:** 2021-11-03

**Authors:** Shao-Lai Zhou, Hao-Yang Xin, Rong-Qi Sun, Zheng-Jun Zhou, Zhi-Qiang Hu, Chu-Bin Luo, Peng-Cheng Wang, Jia Li, Jia Fan, Jian Zhou

**Affiliations:** 1 Department of Liver Surgery and Transplantation, Liver Cancer Institute, Zhongshan Hospital, Fudan University, Shanghai, China; 2 Key Laboratory of Carcinogenesis and Cancer Invasion, Fudan University, Ministry of Education, Shanghai, China; 3 State Key Laboratory of Genetic Engineering, Fudan University, Shanghai, China

## Abstract

**Importance:**

*KRAS* variants are associated with tumor progression; however, the prevalence of *KRAS* variant subtypes and their association with survival and recurrence in patients with intrahepatic cholangiocarcinoma (ICC) after curative resection are largely unknown.

**Objective:**

To explore the prognostic association of *KRAS* variant subtypes with survival and recurrence in patients with ICC.

**Design, Setting, and Participants:**

In this cohort study, patients who underwent curative resection for ICC from January 2009 through December 2016 at a single hospital in China were recruited, and whole-exome sequencing, targeted sequencing, and Sanger sequencing were performed to identify *KRAS* variants. Kaplan-Meier and log-rank tests were used to compare overall survival (OS) and disease-free survival (DFS). Univariate and multivariate analyses were performed using the Cox proportional hazards regression model. Data were analyzed from April 2020 to January 2021.

**Interventions:**

Hepatectomy in patients with ICC.

**Main Outcomes and Measures:**

The association of *KRAS* variant subtypes with OS and DFS.

**Results:**

Of 1024 included patients with ICC, 621 (60.6%) were male, and the mean (SD) age was 59.2 (10.2) years. A total of 14 different subtypes of *KRAS* somatic variants affecting 127 patients (12.4%) were identified. G12D was the most frequent allele in this cohort, accounting for 55 of 127 identified *KRAS* variants (43.3%), followed by G12V (25 [19.7%]), G12C (9 [7.1%]), and G13D (8 [6.3%]). Compared with patients with wild-type *KRAS*, patients with variant *KRAS* were more likely to have high levels of carbohydrate antigen 19-9 (92 of 127 [72.4%] vs 546 of 897 [60.9%]; *P* = .01) and γ-glutamyltransferase (72 of 127 [56.7%] vs 420 of 897 [46.8%]; *P* = .04). Multivariable analysis revealed that G12 *KRAS* variants but not non-G12 *KRAS* variants were independently associated with worse OS (hazard ratio [HR], 1.69; 95% CI, 1.31-2.18; *P* < .001) and DFS (HR, 1.47; 95% CI, 1.16-1.88; *P* = .002). Among the patients with G12 *KRAS* variants, the G12V *KRAS* variant was the strongest prognostic determinant for the worst OS (HR, 3.05; 95% CI, 1.94-4.79; *P* < .001) and DFS (HR, 1.79; 95% CI, 1.13-2.85; *P* = .01).

**Conclusions and Relevance:**

In this cohort study, the distribution of *KRAS* variant subtypes was characterized in a large cohort of patients with ICC from China. The presence of G12 *KRAS* variants but not non-G12 *KRAS* variants was associated with worse survival and increased risk of recurrence. Patients with the G12V variant exhibited the worst outcomes in the whole cohort.

## Key Points

**Question:**

What is the prevalence of *KRAS* variant subtypes and their association with survival and recurrence in patients with surgically treated intrahepatic cholangiocarcinoma (ICC)?

**Findings:**

In this cohort study including 1024 patients, a total of 14 different subtypes of *KRAS* somatic variants affecting 127 patients with ICC (12.4%) were identified, including G12D (43.3%), G12V (19.7%), G12C (7.1%), and G13D (6.3%). G12 *KRAS* variants but not non-G12 *KRAS* variants were independently associated with worse overall and disease-free survival, and the G12V *KRAS* variant was the strongest prognostic determinant for the worst overall and disease-free survival.

**Meaning:**

This cohort study characterized the distribution of *KRAS* variant subtypes in a large cohort of patients with ICC and showed an association with patient outcome.

## Teaser

This cohort study explores the prognostic association of *KRAS* variant subtypes with survival and recurrence in patients with intrahepatic cholangiocarcinoma.

## Introduction

Intrahepatic cholangiocarcinoma (ICC) has an increasing incidence worldwide and is already the second most common primary hepatic malignancy after hepatocellular carcinoma.^1,2^ Advances in diagnostic modalities and clinical screening have made early detection and curative resection possible; however, high relapse rates hinder the long-term survival of patients.^1^ Recently, we have expanded our understanding of the pathogenesis of ICC at the molecular level,^3,4,5,6^ shedding new light on how to treat this malignancy.^1^

The *KRAS protooncogene, GTPase* (*KRAS*; OMIM 190070) gene encodes an oncoprotein involved in key signaling pathways for tumor growth and metastasis.^7^ *KRAS* is affected in nearly one-quarter of a wide spectrum of cancers, predominantly adenocarcinomas, including ICC.^1,7,8^ The distribution of *KRAS* variant subtypes differs across cancer types, with the G12C allele accounting for about 50% of *KRAS* variants in lung cancer and G12D being the most common allele in pancreatic and colorectal cancers.^8^ However, the distribution of *KRAS* variant subtypes in ICC and their association with patient prognosis are largely unknown. Therefore, we investigated the prevalence of *KRAS* variant subtypes and their association with survival and recurrence after curative resection in a large cohort of patients with ICC.

## Methods

### Patients and Follow-up

We enrolled a total of 1024 patients with primary ICC who received curative resection from January 2009 to December 2016 in the Department of Liver Surgical Oncology of Zhongshan Hospital, Fudan University, Shanghai, China, and tissue samples from tumors and matched noncancerous livers were continually collected. Patients receiving palliative procedures or prior interventions (such as transhepatic artery embolization, chemotherapy, or radiotherapy) or with other primary malignancies and inflammatory diseases during the follow-up were excluded from the study. Curative resection was defined as complete resection of tumor nodules, with cancer-free tumor margins shown by histologic examination and resection of regional lymph nodes, including the hilar, hepatoduodenal ligament, and caval lymph nodes, with no cancerous thrombus in the portal vein (main trunk or major branches), hepatic veins, or bile duct.^9^ Patients with further lymph node involvement were considered to have distant metastasis and were excluded from the study. Tumor differentiation was graded histologically according to the Edmondson-Steiner criteria.^10^ Liver function was graded according to the Child-Pugh system. Tumor stage was determined according to the 2017 International Union Against Cancer TNM system. Before surgical operation and tissue sample collection, we obtained oral and written informed consent from each participant, with information such as the use of tissue sample and clinical characteristics for scientific research, which was granted by the Research Ethics Committee of Zhongshan Hospital. For this study, the Research Ethics Committee of Zhongshan Hospital granted ethical approval for the use of human subjects as well as review and approval of this study. We also obtained oral informed consent for inclusion in the study from study participants at the time of follow-up. The clinicopathologic characteristics of the patients are listed in eTable 1 in the Supplement.

**Figure 1.**
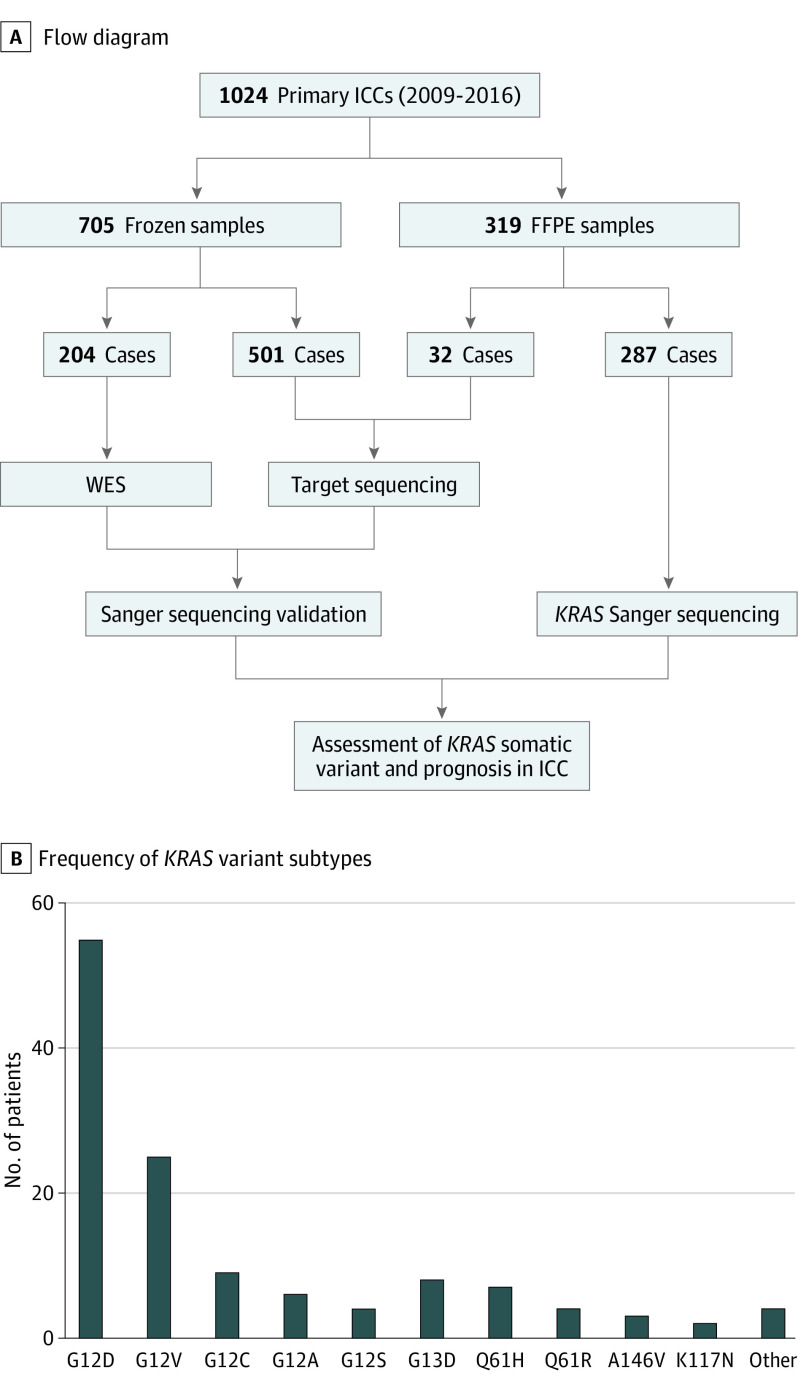
*KRAS* Variant in Intrahepatic Cholangiocarcinoma (ICC) A, Concise flowchart of this study. B, Frequency of *KRAS* variant subtypes in 127 patients with ICC. FFPE indicates formalin fixation and paraffin embedding; WES, whole-exome sequencing.

A concise flowchart of this study is shown in Figure 1A. In these ICCs, 705 cases were frozen samples, and 319 cases were formalin fixation and paraffin embedding (FFPE) samples. A total of 204 ICCs with frozen samples were subjected to whole-exome sequencing, and 501 ICCs with frozen samples and 32 ICCs with FFPE samples were subjected to targeted sequencing. All *KRAS* variants identified through whole-exome sequencing and targeted sequencing were validated by Sanger sequencing. All coding exons of *KRAS* identified to harbor somatic variants were further screened in an additional 287 ICCs (FFPE samples).

The present study includes follow-up data collected through December 2018. The follow-up procedures are described in detail elsewhere.^9,11^ We diagnosed tumor recurrence on the basis of computed tomography scans, magnetic resonance imaging, digital subtraction angiography, and elevated serum carbohydrate antigen 19-9 (CA19-9) level, with or without histological confirmation.^9^ We defined disease-free survival (DFS) as the interval between the surgery and any diagnosis of recurrence (intrahepatic or extrahepatic). We defined overall survival (OS) as the time from the date of surgery until death or the end of follow-up.^9^ The surviving patients were censored at the time of the end of follow-up.

### Statistical Analysis

Statistical analyses were conducted using R version 3.6.2 (The R Foundation) or using SPSS version 16.0 (IBM). The χ^2^ or Fisher exact tests were used to compare categorical data. The Kaplan-Meier method was used to calculate the OS and DFS. Differences were analyzed by the log-rank test. Univariate and multivariate analyses were performed using the Cox proportional hazards regression model. All tests were 2-sided, and *P* values less than .05 were considered to be statistically significant. Other information about the methods is available in eMethods 1 to 4 in the Supplement.

## Results

### *KRAS* Variant Subtypes

Of 1024 patients recruited in this study (621 men [60.6%] and 403 women [39.4%]; mean [SD] age, 59.2 [10.2] years), a total of 14 different subtypes of *KRAS* somatic variant affecting 127 patients with ICC (12.4%) were identified, including 5 types of G12* variant, 1 type of G13* variant, and 3 types of Q61* variant. In addition, we identified several uncommon *KRAS* alleles, including A146V, K117N, and others (Figure 1B; eTable 2 in the Supplement). A total of 55 patients (43.3%) demonstrated a G12D genotype, which was the most frequent allele in our cohort, followed by the G12V allele (25 patients [19.7%]), the G12C allele (9 [7.1%]), and the G13D allele (8 [6.3%]).

### Clinical Characteristics

The clinical characteristics of the patients with wild-type *KRAS* (wt*KRAS*) and variant *KRAS* (vt*KRAS*) genotypes are presented in eTable 3 in the Supplement. Compared with patients with wt*KRAS*, patients with vt*KRAS* were more likely to have high levels of CA19-9 (92 of 127 [72.4%] vs 546 of 897 [60.9%]; *P* = .01) and γ-glutamyltransferase (72 of 127 [56.7%] vs 420 of 897 [46.8%]; *P* = .04). Among patients with vt*KRAS*, patients with G12 *KRAS* variants were more likely than those with non-G12 *KRAS* variants to have elevated γ-glutamyltransferase levels (63 of 99 [64%] vs 9 of 28 [32%]; *P* = .003), large tumor size (57 of 99 [58%] vs 8 of 28 [29%]; *P* = .007), and lymphatic metastasis (19 of 99 [19%] vs 0; *P* = .01).

### Survival Analysis

**Figure 2.**
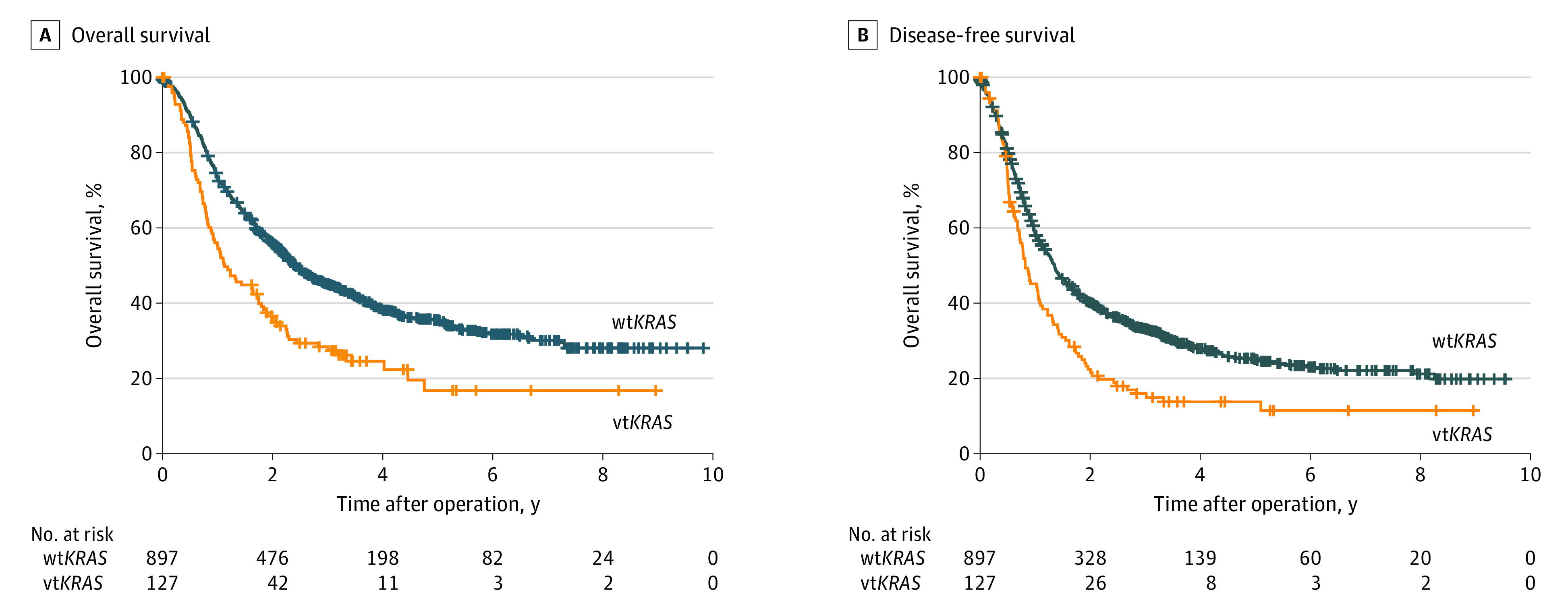
Association of *KRAS* Variants With Patient Outcome A, Kaplan-Meier survival analysis showing overall survival based on wild-type *KRAS* (wt*KRAS*) and variant *KRAS* (vt*KRAS*). B, Kaplan-Meier survival analysis showing disease-free survival based on wt*KRAS* and vt*KRAS*.

**Figure 3.**
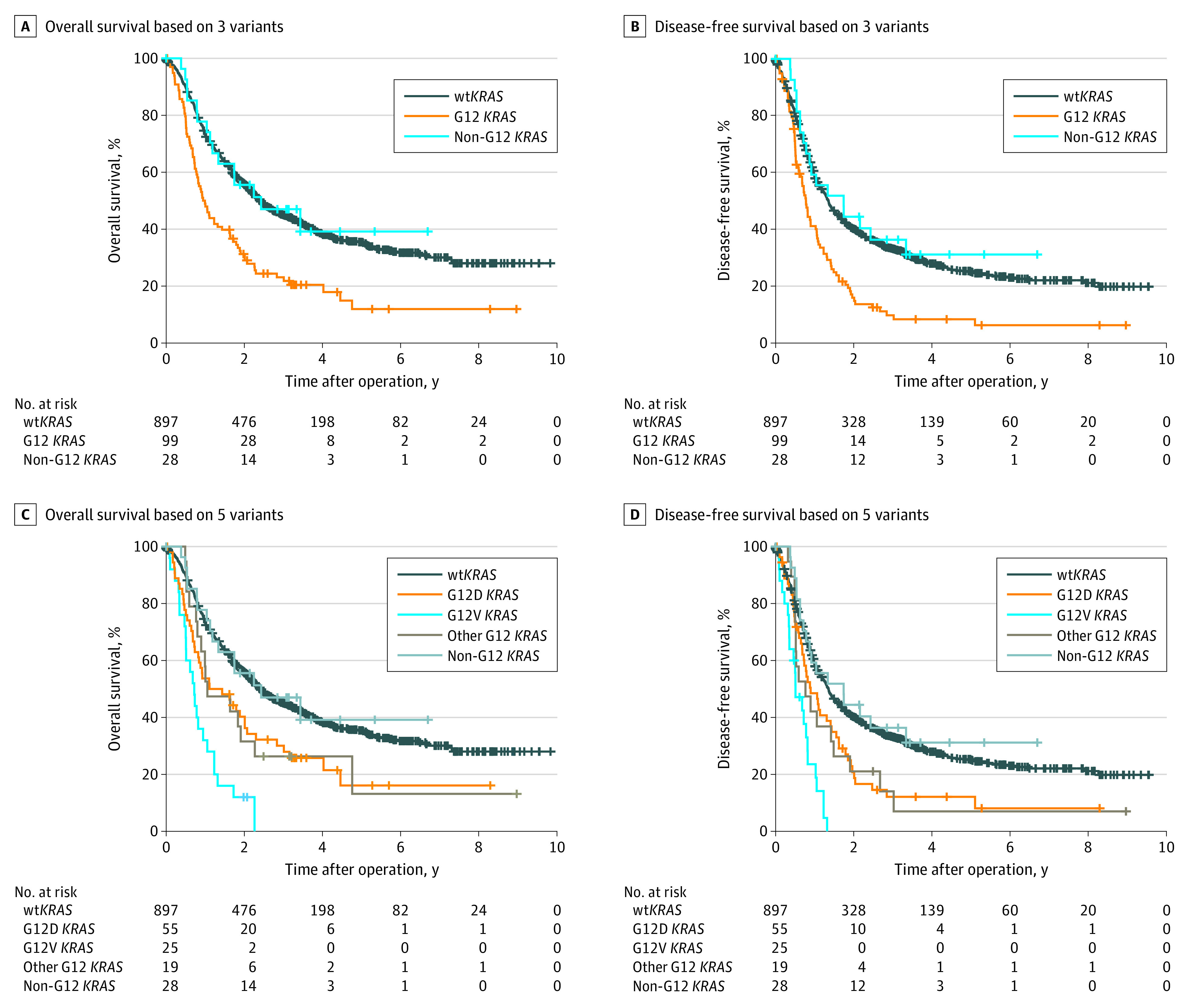
Association of *KRAS* Variant Subtype With Patient Outcome A, Kaplan-Meier survival analysis showing overall survival based on wild-type *KRAS* (wt*KRAS*), G12 *KRAS*, and non-G12 *KRAS* variants. B, Kaplan-Meier survival analysis showing disease-free survival based on wt*KRAS*, G12 *KRAS*, and non-G12 *KRAS* variants. C, Kaplan-Meier survival analysis showing overall survival based on wt*KRAS*, G12D *KRAS*, G12V *KRAS*, other G12 *KRAS*, and non-G12 *KRAS* variants. D, Kaplan-Meier survival analysis showing disease-free survival based on wt*KRAS*, G12D *KRAS*, G12V *KRAS*, other G12 *KRAS*, and non-G12 *KRAS* variants.

At a median (IQR) follow-up of 53.4 (36.8-74.3) months, 641 of 1024 patients with ICC (62.6%) had died, and 42 patients (4.1%) were lost to follow-up. Across all patients considered, *KRAS* status was significantly associated with OS (hazard ratio [HR], 1.70; 95% CI, 1.36-2.11; *P* < .001) and DFS (HR, 1.54; 95% CI, 1.25-1.90; *P* < .001) (Table; Figure 2A and B). Among the patients with vt*KRAS*, G12 *KRAS* variants were significantly associated with inferior OS (median OS, 11.3 [95% CI, 8.3-14.3] months vs 29.1 [95% CI, 5.4-52.9] months; *P* = .009) and DFS (median DFS, 9.3 [95% CI, 7.8-10.8] months vs 20.9 [95% CI, 6.5-35.2] months; *P* = .003) compared with those with non-G12 *KRAS* variants. Patients with non-G12 *KRAS* variants had median OS and DFS comparable with patients with wt*KRAS* status (median OS, 28.9 [95% CI, 25.2-32.6] months; median DFS, 16.2 [95% CI, 14.3-18.2] months) (Figure 3A and B). In the univariate analysis, G12 *KRAS* variants but not non-G12 *KRAS* variants were significantly prognostic for OS (HR, 2.00; 95% CI, 1.58-2.53; *P* < .001) and DFS (HR, 1.83; 95% CI, 1.48-2.29; *P* < .001) (Table). In multivariable analysis, G12 *KRAS* variants remained significantly associated with poor OS (HR, 1.69; 95% CI, 1.31-2.18; *P* < .001) and DFS (HR, 1.47; 95% CI, 1.16-1.88; *P* = .002) (Table).

**Table.** Univariate and Multivariate Analyses of Prognostic Factors Among 1024 Patients With Intrahepatic Cholangiocarcinoma

Variable	Univariate analyses		Multivariate analyses	
	HR (95% CI)	*P* value	HR (95% CI)	*P* value
Overall survival				
Age (>50 vs ≤50 y)	1.24 (1.01-1.51)	.04	1.30 (1.05-1.61)	.02
Sex (male vs female)	1.20 (1.02-1.41)	.03	1.24 (1.05-1.46)	.01
HBsAg (positive vs negative)	0.73 (0.61-0.87)	<.001	0.78 (0.65-0.94)	.007
CA19-9, U/mL (>36 vs ≤36)	1.63 (1.38-1.92)	<.001	1.29 (1.09-1.54)	.004
GGT, U/L (>54 vs ≤54)	1.78 (1.51-2.09)	<.001	1.37 (1.15-1.62)	<.001
Liver cirrhosis (yes vs no)	0.88 (0.73-1.05)	.16	NA	NA
Tumor size (>5 vs ≤5 cm)	1.66 (1.42-1.94)	<.001	1.31 (1.10-1.56)	.003
Tumors (multiple vs single)	2.13 (1.79-2.53)	<.000	1.80 (1.49-2.16)	<.001
Microvascular/bile duct invasion (yes vs no)	1.66 (1.38-1.98)	<.001	1.33 (1.09-1.62)	.005
Lymphatic metastasis (yes vs no)	2.83 (2.32-3.45)	<.001	2.16 (1.74-2.67)	<.001
Tumor encapsulation (none vs complete)	1.29 (1.01-1.65)	.04	1.10 (0.85-1.42)	.47
Tumor differentiation (III or IV vs I or II)	1.41 (1.21-1.65)	<.001	1.38 (1.17-1.63)	<.001
* KRAS *subtype				
VT vs WT	1.70 (1.36-2.11)	<.001	1.55 (1.22-1.95)	<.001
Non-G12 VT vs WT	0.95 (0.57-1.58)	.84	NA	NA
All G12 VT vs WT	2.00 (1.58-2.53)	<.001	1.69 (1.31-2.18)	<.001
G12D VT vs WT	1.67 (1.22-2.30)	.001	1.36 (0.97-1.91)	.08
G12V VT vs WT	3.89 (2.55-5.93)	<.001	3.05 (1.94-4.79)	<.001
Other G12 VT vs WT	1.67 (1.00-2.78)	.05	1.66 (0.91-3.04)	.10
Disease-free survival				
Age (>50 vs ≤50 y)	0.98 (0.81-1.19)	.78	NA	NA
Sex (male vs female)	1.21 (1.05-1.41)	.01	1.24 (1.06-1.45)	.008
HBsAg (positive vs negative)	0.90 (0.77-1.06)	.19	NA	NA
CA19-9, U/mL (>36 vs ≤36)	1.48 (1.27-1.73)	<.001	1.21 (1.03-1.42)	.02
GGT, U/L (>54 vs ≤54)	1.63 (1.40-1.89)	<.001	1.35 (1.16-1.58)	<.001
Liver cirrhosis (yes vs no)	0.97 (0.82-1.15)	.75	NA	NA
Tumor size (>5 vs ≤5 cm)	1.75 (1.51-2.03)	<.001	1.46 (1.24-1.72)	<.001
Tumors (multiple vs single)	1.88 (1.59-2.22)	<.001	1.55 (1.29-1.85)	<.001
Microvascular/bile duct invasion (yes vs no)	1.55 (1.30-1.85)	<.001	1.24 (1.02-1.50)	.03
Lymphatic metastasis (yes vs no)	2.28 (1.88-2.77)	<.001	1.82 (1.48-2.23)	<.001
Tumor encapsulation (none vs complete)	1.18 (0.95-1.48)	.14	NA	NA
Tumor differentiation (III or IV vs I or II)	1.24 (1.08-1.44)	.003	1.25 (1.07-1.46)	.004
* KRAS *subtype				
VT vs WT	1.54 (1.25-1.90)	<.001	1.31 (1.05-1.64)	.02
Non-G12 VT vs WT	0.89 (0.55-1.42)	.61	NA	NA
All G12 VT vs WT	1.83 (1.48-2.29)	<.001	1.47 (1.16-1.88)	.002
G12D VT vs WT	1.55 (1.15-2.09)	.004	1.28 (0.94-1.75)	.12
G12V VT vs WT	3.29 (2.16-5.01)	<.001	1.79 (1.13-2.85)	.01
Other G12 VT vs WT	1.63 (1.01-2.64)	.05	1.80 (1.03-3.13)	.04

Abbreviations: CA19-9, carbohydrate antigen 19-9; GGT, γ-glutamyltransferase; HBsAg, hepatitis B surface antigen; HR, hazard ratio; NA, not applicable; VT, variant type; WT, wild type.

SI conversion factor: To convert GGT to μkat/L, multiply by 0.0167.

When we divided the G12 *KRAS* variants into G12D *KRAS*, G12V *KRAS*, and other G12 *KRAS* subtypes, we observed that the median OS and DFS were comparable between patients with the G12D *KRAS* allele and with other G12 *KRAS* alleles (OS, 13.3 [95% CI, 3.0-23.6] months vs 12.6 [95% CI, 1.7-23.6] months; DFS, 10.8 [95% CI, 6.5-15.0] months vs 9.2 [95% CI, 2.9-15.5] months) (Figure 3C and D), both of which had shorter OS and DFS than patients with wt*KRAS*. Moreover, patients with the G12V *KRAS* allele exhibited even worse median OS (8.6 months; 95% CI, 6.3-10.9) and DFS (6.1 months; 95% CI, 2.9-9.3) than patients with the G12D *KRAS* allele or with other G12 *KRAS* alleles. In the univariate analysis, the G12D *KRAS* allele and other G12 *KRAS* alleles were associated with inferior OS (G12D *KRAS *vs wt*KRAS*: HR, 1.67; 95% CI, 1.22-2.30; *P* = .001; other G12 *KRAS *vs wt*KRAS*: HR, 1.67; 95% CI, 1.00-2.78; *P* = .05) and DFS (G12D *KRAS *vs wt*KRAS*: HR, 1.55; 95% CI, 1.15-2.09; *P* = .004; other G12 *KRAS *vs wt*KRAS*: HR, 1.63; 95% CI, 1.01-2.64; *P* = .047). Furthermore, the G12V *KRAS* allele was the strongest prognostic determinant for worst OS (G12V *KRAS *vs wt*KRAS*: HR, 3.89; 95% CI, 2.55-5.93; *P* < .001) and DFS (G12V *KRAS *vs wt*KRAS*: HR, 3.29; 95% CI, 2.16-5.01; *P* < .001). Multivariate analyses also revealed that the G12V *KRAS* allele was independently associated with OS (HR, 3.05; 95% CI, 1.94-4.79; *P* < .001) and DFS (HR, 1.79; 95% CI, 1.13-2.85; *P* = .01) (Table).

## Discussion

We analyzed *KRAS* variant subtypes and their association with ICC characteristics and prognosis in, to our knowledge, the largest cohort of patients with ICC that has been analyzed to date. The frequency of *KRAS* variation in our cohort was 12.4%, which is higher than in The Cancer Genome Atlas and Memorial Sloan Kettering Integrated Mutation Profiling of Actionable Cancer Targets cohorts (3.3% and 6.3%, respectively),^12,13^ possibly due to differences in racial or etiological factors. Until now, to our knowledge, no study has investigated *KRAS* variant subtypes in ICC, perhaps due to the limited sample size. The distribution of *KRAS* variant subtypes in our cohort was different than that in other types of cancer, such as lung adenocarcinoma, colon adenocarcinoma, and pancreatic ductal adenocarcinoma (eTable 4 in the Supplement).^8^ We propose that the *KRAS* variant patterns are a product, at least in part, of selection during tumor initiation for an ideal level of signaling, which is shaped by the variations in the function of particular *KRAS* variants, KRAS protein levels, and cellular responses to oncogenic KRAS.^8^

The presence of *KRAS* variation was a negative independent prognostic factor for survival and recurrence in patients with ICC, which is consistent with previous findings.^5,6^ We found that this prognostic value was mainly owing to G12 *KRAS* variants because non-G12 *KRAS* variants demonstrated no significant association with prognosis. Furthermore, our results indicated that different G12 *KRAS* variants may have distinct associations with survival. Specifically, the G12V *KRAS* variant was associated with the worst prognosis, whereas the G12D *KRAS* variant and other G12 *KRAS* variants were associated with poorer prognosis than the non-G12 *KRAS* variant but better prognosis than the G12V *KRAS* variant. Although this was a single-center retrospective study, the uniform standard of care and identical follow-up support the accuracy of the survival analysis.

In addition to being associated with prognosis after curative resection among patients with ICC, we found that G12 *KRAS* variants but not non-G12 *KRAS* variants were associated with lymphatic metastasis, especially G12V *KRAS* variants, which were enriched in patients with lymphatic metastasis (9 [6.3%] vs 16 [1.8%]; *P* = .005) (eTable 5 in the Supplement). Several studies have reported prognostic factors for ICC, with lymphatic metastasis confirmed to be one of the most significant independent indicators,^14,15^ which was also confirmed in our study. Thus, we propose that G12V *KRAS* variant may contribute to the worst prognosis in ICC by mediating tumor lymphatic metastasis. These patients should be more carefully monitored during perioperative period and after curative resection.

Recent advances in medicinal chemistry, which are involved with design, chemical synthesis, and development for market of pharmaceutical agents or bioactive molecules, have identified inhibitors targeting G12C *KRAS* variants, which are mainly found in lung adenocarcinomas.^7^ Our results suggest that in patients with ICC, the G12D *KRAS* variant has the greatest prevalence, whereas the G12V *KRAS* variant is the strongest prognostic determinant and therefore probably the most oncogenic of the *KRAS* variants. These results warrant further pursuit of specific inhibitors of G12D *KRAS* and G12V *KRAS* oncoproteins in precision oncology for patients with ICC.

### Limitations

This study has limitations. Our analysis is limited by the retrospective nature of the study and its exclusive focus on patients with surgically resectable disease. As such, a degree of selection bias was largely unavoidable. Second, this study came from China, and further research is warranted for international validation.

## Conclusions

This cohort study characterized the distribution *KRAS* variant subtypes in a large ICC cohort. The presence of G12 *KRAS* variants but not non-G12 *KRAS* variants was associated with poor survival and increased risk of recurrence. Patients with the G12V variant had significantly worse prognosis than patients with any other *KRAS* alleles across the entire cohort.

## Notes

Supplement. **eMethods 1.** DNA preparation, DNA capture, and sequencing. **eMethods 2.** Whole-exome and targeted sequencing. **eMethods 3.** Reads mapping and detection of somatic genetic alterations. **eMethods 4.** Sanger sequencing. **eTable 1.** Clinicopathologic characteristics of patients with intrahepatic cholangiocarcinoma (n=1024). **eTable 2.** 127 KRAS somatic mutation identified across all ICC patients. **eTable 3.** Correlation between KRAS mutation status and clinicopathologic characteristics. **eTable 4.** Top 5 amino acid substitutions and frequencies with KRAS in 3 other types of cancers commonly associated with KRAS mutations and ICC. **eTable 5.** Correlation between G12 KRAS mutations subtypes and microvascular/bile duct invasion or lymphatic metastasis in 1024 ICC patients.
eReferences.
