# Supplementary material for: Association of KRAS Variant Subtypes With Survival and Recurrence in Patients With Surgically Treated Intrahepatic Cholangiocarcinoma
Source: JAMA Surg. 2021 Nov 3;157(1):1–7. doi: 10.1001/jamasurg.2021.5679 (PMC8567187; doi:10.1001/jamasurg.2021.5679)
Supplement: Supplement. — eMethods 1. DNA preparation, DNA capture, and sequencing. eMethods 2. Whole-exome and targeted sequencing. eMethods 3. Reads mapping and detection of somatic genetic alterations. eMethods 4. Sanger sequencing. eTable 1. Clinicopathologic characteristics of patients with intrahepatic cholangiocarcinoma (n=1024). eTable 2. 127 KRAS somatic mutation identified across all ICC patients. eTable 3. Correlation between KRAS mutation status and clinicopathologic characteristics. eTable 4. Top 5 amino acid substitutions and frequencies with KRAS in 3 other types of cancers commonly associated with KRAS mutations and ICC. eTable 5. Correlation between G12 KRAS mutations subtypes and microvascular/bile duct invasion or lymphatic metastasis in 1024 ICC patients. eReferences. [file jamasurg-e215679-s001.pdf]

Supplemental Online Content

Zhou S-L, Xin H-Y, Sun R-Q, et al. Association of *KRAS* variant subtypes with survival and recurrence in patients with surgically treated intrahepatic cholangiocarcinoma. *JAMA Surg*. Published online November 3, 2021. doi:10.1001/jamasurg.2021.5679

eMethods 1. DNA preparation, DNA capture, and sequencing
eMethods 2. Whole-exome and targeted sequencing
eMethods 3. Reads mapping and detection of somatic genetic alterations
eMethods 4. Sanger sequencing
eTable 1. Clinicopathologic characteristics of patients with intrahepatic cholangiocarcinoma (n=1024)
eTable 2. 127 *KRAS* somatic mutation identified across all ICC patients
eTable 3. Correlation between *KRAS* mutation status and clinicopathologic characteristics
eTable 4. Top 5 amino acid substitutions and frequencies with KRAS in 3 other types of cancers commonly associated with *KRAS* mutations and ICC
eTable 5. Correlation between G12 *KRAS* mutations subtypes and microvascular/bile duct invasion or lymphatic metastasis in 1024 ICC patients
eReferences.

This supplementary material has been provided by the authors to give readers additional information about their work.

© 2021 Zhou S-L et al. *JAMA Surgery*.

eMethods 1. DNA preparation, DNA capture, and sequencing

Snap frozen tissue samples from tumor and matched non-cancerous liver were obtained and embedded in OCT compound, sectioned by a cryostat, and stained by hematoxylin and eosin. We performed macrodissection to enrich the tumor fraction relative to the dominant stromal component and other normal cells. For FFPE samples, samples were cut into 5–10 consecutive 10-μm sections for laser capture microdissection (LCM). DNA was extracted using the QIAamp DNA Micro Kit (Qiagen) according to the manufacturer's protocol. Preparation of sequencing libraries and DNA capture methods were carried out according to the manufacturer's protocols.

© 2021 Zhou S-L et al. *JAMA Surgery*.

eMethods 2. Whole-exome and targeted sequencing

For WES, snap-frozen tissue samples from 204 primary tumors and matched non-cancerous liver from 204 ICC patients were subjected to whole exome sequencing. Sequencing libraries were constructed using a modification of the KAPA Library Preparation Kit.[3] Libraries were combined into pools for solution phase hybridization using the Agilent SureDesign Human All Exon V6, and then sent for sequencing to generate 2×150 bp paired-end reads using Novaseq platform (Illumina, Inc.). For targeted sequencing, 501 frozen tumor samples from 501 ICC patients and 32 FFPE tumor samples from 32 ICC patients were subjected to targeted sequencing. We used a multiplex polymerase chain reaction-based next-generation sequencing platform, which containing 12 amplicons from genomic DNA for regions covering all coding exons in *KRAS*.

© 2021 Zhou S-L et al. *JAMA Surgery*.

eMethods 3. Reads mapping and detection of somatic genetic alterations

Valid sequencing data was mapped to the reference human genome (UCSC hg19) using the Burrows-Wheeler Aligner (BWA)[4] software to get the original mapping results stored in BAM format. We performed local realignment of the original BAM alignment using the GATK2[5] and then marked duplicate reads using Sambamba[6].

Somatic SNVs were detected by muTect,[7] and somatic indels were detected by Strelka.[8] High-confidence somatic mutations were called if the following criteria were met: (1) both the tumor and normal samples were covered sufficiently (≥10×) at the genomic level; (2) the variants were supported by at least 5% of the total reads in the tumor and less than 1% of the total reads in the normal tissue; (3) the variants were supported by at least three reads in the tumor. ANNOVAR[4] was performed to do annotation for the Variant Call Format obtained in the previous effort. Somatic SNVs and indels that were referenced in the 1000 Genomes Project with a minor allele frequency over 1% or located in segmental duplications were removed along with common SNPs.

© 2021 Zhou S-L et al. *JAMA Surgery*.

eMethods 4. Sanger sequencing

All *KRAS* mutations identified through WES and targeted sequencing were validated by Sanger sequencing. All coding exons of *KRAS* identified to harbor somatic mutations were further screened in an additional 287 ICCs (FFPE samples). Sanger sequencing primers were designed using the Primer3 software (http://frodo.wi.mit.edu/). All mutations identified in tumors were confirmed by independent PCR and Sanger sequencing in the specific tumors and their paired normal tissues to determine their somatic nature.

© 2021 Zhou S-L et al. *JAMA Surgery*.

eTable 1. Clinicopathologic characteristics of patients with intrahepatic cholangiocarcinoma (n=1024)

Characteristics	Number (%)
Age, year (≤50 versus >50)	200/824 (19.5/80.5)
Sex (female versus male)	403/621 (39.4/60.6)
HBsAg (negative versus positive)	721/303 (70.4/29.6)
CA199(≤36 versus >36)	386/638 (37.7/62.3)
GGT,U/L (≤54 versus>54)	532/492 (52.0/48.0)
Liver cirrhosis (no versus yes)	761/263 (74.3/25.7)
Tumor size,cm(≤5 versus>5)	488/536 (47.7/52.3)
Tumor number (single versus multiple)	793/231 (77.4/22.6)
Microvascular/bile duct invasion (no versus yes)	819/205 (80.0/20.0)
Lymphatic metastasis (no versus yes)	880/144 (85.9/14.1)
Tumor encapsulation (complete versus none)	130/894 (12.7/87.3)
Tumor differentiation (I+II versus III+IV)	496/528 (48.4/51.6)

Abreviations: AFP, alpha-fetoprotein; GGT, gamma glutamyl transferase; CA 19-9, carbohydrate antigen 19-9.

© 2021 Zhou S-L et al. *JAMA Surgery*.

eTable 2. 127 *KRAS* somatic mutation identified across all ICC patients

Sample	*KRAS* mutation	Sample	*KRAS* mutation	Sample	*KRAS* mutation
ICC-6	G12D	ICC-405	G12V	ICC-791	G13D
ICC-29	A146V	ICC-422	G12V	ICC-793	G13D
ICC-32	D47N	ICC-431	G13D	ICC-801	G12D
ICC-50	G12D	ICC-464	G12D	ICC-805	G12D
ICC-62	G12C	ICC-468	G12D	ICC-815	G12S
ICC-63	G12V	ICC-487	G12D	ICC-816	G12V
ICC-68	G12D	ICC-496	G12V	ICC-830	G12V
ICC-77	Q61H	ICC-501	G12V	ICC-848	K117N
ICC-87	G12D	ICC-503	G13D	ICC-853	Q61R
ICC-90	G12V	ICC-513	Q61H	ICC-854	G12V
ICC-91	G12D	ICC-518	G12D	ICC-859	G12V
ICC-97	G12D	ICC-527	G12D	ICC-861	G12D
ICC-99	G12V	ICC-532	G12D	ICC-874	G12D
ICC-102	G12V	ICC-536	N86D	ICC-878	G12V
ICC-104	G12C	ICC-542	G12D	ICC-886	G12V
ICC-108	G12D	ICC-545	G12S	ICC-892	G12V
ICC-132	G13D	ICC-547	T50P	ICC-895	G12D
ICC-146	G12D	ICC-554	Q61R	ICC-901	G12V
ICC-158	G12D	ICC-565	G12D	ICC-911	G12C
ICC-160	G12D	ICC-573	G12S	ICC-913	G12D
ICC-167	G12D	ICC-580	G12V	ICC-916	A146V
ICC-175	Q61L	ICC-585	G12V	ICC-924	G12V
ICC-190	G12D	ICC-603	G12V	ICC-925	G12V
ICC-207	G12V	ICC-626	G12D	ICC-928	G12D
ICC-213	G12C	ICC-640	K117N	ICC-933	G12D
ICC-246	G12D	ICC-645	Q61H	ICC-937	G12C
ICC-253	G12D	ICC-649	G12D	ICC-941	G12C
ICC-257	G12D	ICC-665	G12C	ICC-946	G12A
ICC-260	G12C	ICC-666	G12A	ICC-954	G12D
ICC-287	Q61H	ICC-669	G12D	ICC-957	G12C
ICC-293	G12S	ICC-680	G12D	ICC-965	G12D
ICC-304	G12D	ICC-698	G12D	ICC-980	A146V
ICC-308	G13D	ICC-701	G12V	ICC-981	G12D
ICC-312	G12D	ICC-708	G12D	ICC-982	G12D
ICC-320	G12D	ICC-709	Q61R	ICC-984	Q61H
ICC-331	G12D	ICC-711	G12A	ICC-987	G12D
ICC-334	G12V	ICC-712	G12A	ICC-990	Q61H
ICC-352	G12D	ICC-714	G12A	ICC-994	G12D
ICC-354	G12D	ICC-742	G12D	ICC-1000	Q61R
ICC-356	G12D	ICC-756	G12D	ICC-1016	G12A
ICC-382	G12V	ICC-765	G12D	ICC-1023	Q61H

© 2021 Zhou S-L et al. *JAMA Surgery*.

ICC-391	G13D	ICC-772	G13D		
ICC-395	G12D	ICC-784	G12D		

© 2021 Zhou S-L et al. *JAMA Surgery*.

eTable 3. Correlation between *KRAS* mutation status and clinicopathologic characteristics

Clinicopathological indexes		No. of Patients					
		All patients	*KRAS* WT	*KRAS* G12MT	*KRAS* non-G12MT	Test 1 *P**	Test 2 *P*†
Age(year)	≤50	200	181	15	4	0.17	0.91
	>50	824	716	84	24		
Sex	Female	403	351	40	12	0.70	0.82
	Male	621	546	59	16		
HBsAg	Negative	721	625	78	18	0.17	0.12
	Positive	303	272	21	10		
CA199 (U/mL)	≤20	386	351	27	8	0.01	0.89
	>20	638	546	72	20		
GGT (U/L)	≤54	532	477	36	19	0.04	0.003
	>54	492	420	63	9		
Liver cirrhosis	No	761	666	74	21	0.89	0.98
	yes	263	231	25	7		
Tumor size(cm)	≤5	488	426	42	20	0.78	0.007
	>5	536	471	57	8		
Tumor number	Single	793	701	70	22	0.15	0.41
	Multiple	231	196	29	6		
Vascular/bile duct invasion	absence	819	723	73	23	0.19	0.36
	present	205	174	26	5		
Lymphatic metastasis	absence	880	772	80	28	0.76	0.01
	present	144	125	19	0		
Tumor encapsulation	complete	130	120	8	2	0.08	0.87
	none	894	777	91	26		
Tumor differentiation	I+II	496	439	45	12	0.39	0.81
	III+IV	528	458	54	16		

Abbreviations: GGT, gamma glutamyl transferase; CA 19-9, carbohydrate antigen 19-9

© 2021 Zhou S-L et al. *JAMA Surgery*.

*Test 1 *P* value: two-sided *P* value for test of differences in factor means between *KRAS* MT v *KRAS* WT

†Test 2 *P* value: two-sided *P* value for test of differences in factor means between *KRAS* G12MT v *KRAS* non-G12MT.

© 2021 Zhou S-L et al. *JAMA Surgery*.

eTable 4. Top 5 amino acid substitutions and frequencies with KRAS in 3 other types of cancers commonly associated with *KRAS* mutations and ICC

**PDAC**		**Colon adenocarcinoma**		**LUAD**		**ICC (this study)**	
G12D	33.80%	G12D	10.30%	G12C	6.70%	G12D	5.40%
G12V	22.20%	G12V	6.30%	G12V	4.60%	G12V	2.40%
G12R	9.00%	G13D	5.80%	G12D	3.60%	G12C	0.90%
G12C	1.70%	G12C	2.60%	G12A	1.80%	G13D	0.80%
G12S	1.20%	G12S	1.80%	G13D	0.50%	Q61H	0.70%

Abbreviations: LUAD, lung adenocarcinoma;

PDAC, pancreatic ductal adenocarcinoma;

ICC, intrahepatic cholangiocarcinoma

© 2021 Zhou S-L et al. *JAMA Surgery*.

eTable 5. Correlation between G12 *KRAS* mutations subtypes and microvascular/bile duct invasion or lymphatic metastasis in 1024 ICC patients

***KRAS* mutation status**		**Microvascular/bile duct invasion**		***P***	**Lymphatic metastasis**		***P***
		**No**	**Yes**		**No**	**Yes**	
G12 MT	Absent	746	179	0.10	800	125	0.12
	Present	73	26		80	19	
G12D MT	Absent	779	190	0.17	832	137	0.77
	Present	40	15		48	7	
G12V MT	Absent	800	199	0.62	864	135	0.005
	Present	19	6		16	9	
Other G12 MT	Absent	805	200	0.56	864	141	1.00
	Present	14	5		16	3	

© 2021 Zhou S-L et al. *JAMA Surgery*.

eReferences.

1. Zhou Z, Wang P, Sun R, et al. Tumor-associated neutrophils and macrophages interaction contributes to intrahepatic cholangiocarcinoma progression by activating STAT3. *Journal for immunotherapy of cancer.* Mar 2021;9(3).
2. Wittekind C. [Pitfalls in the classification of liver tumors]. *Pathologe.* Jul 2006;27(4):289-293.
3. Zhou SL, Zhou ZJ, Hu ZQ, et al. Genomic sequencing identifies WNK2 as a driver in hepatocellular carcinoma and a risk factor for early recurrence. *J Hepatol.* Dec 2019;71(6):1152-1163.
4. Li H, Durbin R. Fast and accurate short read alignment with Burrows-Wheeler transform. *Bioinformatics.* Jul 15 2009;25(14):1754-1760.
5. McKenna A, Hanna M, Banks E, et al. The Genome Analysis Toolkit: a MapReduce framework for analyzing next-generation DNA sequencing data. *Genome Res.* Sep 2010;20(9):1297-1303.
6. Tarasov A, Vilella AJ, Cuppen E, Nijman IJ, Prins P. Sambamba: fast processing of NGS alignment formats. *Bioinformatics.* Jun 15 2015;31(12):2032-2034.
7. Cibulskis K, Lawrence MS, Carter SL, et al. Sensitive detection of somatic point mutations in impure and heterogeneous cancer samples. *Nat Biotechnol.* Mar 2013;31(3):213-219.
8. Saunders CT, Wong WS, Swamy S, Becq J, Murray LJ, Cheetham RK. Strelka: accurate somatic small-variant calling from sequenced tumor-normal sample pairs. *Bioinformatics.* Jul 15 2012;28(14):1811-1817.

© 2021 Zhou S-L et al. *JAMA Surgery*.
